# Do partner handholding effects linger? Order effects of handholding support on mechanical hypersensitivity in women

**DOI:** 10.1097/PR9.0000000000001464

**Published:** 2026-07-17

**Authors:** Ketan Prafull Jaltare, Lisa Sanfrinnon, Diana M. Torta

**Affiliations:** a Health Psychology, Faculty of Psychology and Educational Sciences, KU Leuven, Leuven, Belgium; b Pain Focus Group, Leuven Brain Institute, KU Leuven, Leuven, Belgium

**Keywords:** Pain, Hyperalgesia, Mechanical hypersensitivity, Social support, Order effects, Middle frequency stimulation

## Abstract

**Introduction:**

Social support, such as handholding from a romantic partner, can reduce pain and physiological responses to noxious stimuli. We previously demonstrated that handholding during the induction of secondary mechanical hypersensitivity via electrical stimulation reduces the spatial spread of hypersensitivity. However, real-life social contexts are dynamic, and it remains unclear whether such support has lingering effects after its withdrawal or whether prior support buffers against subsequent absence.

**Objectives:**

In this preregistered study, we expanded our earlier sample (N = 37 couples) by adding 28 couples (total N = 65) to examine the effects of partner handholding and condition order on mechanical hypersensitivity.

**Methods:**

Healthy women underwent 2 sessions (support and alone), which involved romantic partner handholding during middle frequency stimulation (MFS) of the volar forearm. We measured the perceived intensity, unpleasantness, and spatial spread of hypersensitivity before and after MFS.

**Results:**

Handholding significantly reduced both vertical and horizontal spread of hypersensitivity, regardless of condition order. Subjective ratings of pain intensity and unpleasantness revealed a complex interaction with condition order. Elevated baseline mechanical sensitivity and MFS ratings were observed when handholding was provided first, while receiving handholding after having been alone yielded the lowest ratings, consistent with safety signaling or relief effects. The post-MFS increase in ratings was inversely related to baseline sensitivity, resulting in no significant differences.

**Conclusion:**

Our findings suggest that although the modulation of hypersensitivity spread by handholding is robust and order-independent, processes related to expectation and habituation shape subjective reports of hypersensitivity and their modulation by handholding.

## Abstract 2

Supplemental Digital Content is Available in the Text.

Partner handholding reduced experimentally induced hypersensitivity spread irrespective of order; subjective pain ratings differed depending on prior support.

## 1. Introduction

Handholding from a romantic partner during the administration of a pain-eliciting stimulus attenuates pain perception and physiological responses to nociceptive input.^13,14,24,30^ We have previously shown that receiving social support during the induction of mechanical hypersensitivity by electrical stimulation buffered against its development in healthy volunteers,^17,18^ reducing the spread of mechanical hypersensitivity.^17^ This is important, as mechanical hypersensitivity is considered a perceptual correlate of activity-induced plasticity in the nociceptive system, which may contribute to the persistence of pain in clinical populations,^2,48^ although its role is debated.^44^ However, such experimental manipulations necessarily impose a static social context that may not reflect the dynamic nature of real-world social interactions. In everyday life, the availability of social support can fluctuate from moment to moment, and the ability to update one's beliefs about the supportive or unsupportive nature of the social context may be critical in determining its influence on pain.^5,37,38^ For instance, the impact of newly available support after an unsupportive context may differ from the impact of losing support after having had it. Psychological factors, such as the negativity bias,^31,39^ may further shape this process, making individuals more likely to maintain negative expectations or to more readily update from positive to negative interpretations than from negative to positive. Although previous studies have generally counterbalanced conditions when examining the effects of social support,^10,20,25,32,49^ only one study to our knowledge has explicitly modelled order effects,^30^ finding no effects. However, this study examined whether the sequence of delivery of different forms of social support influenced pain perception, rather than the change from a supportive to an unsupportive/alone context and vice versa. It remains unclear whether support's benefits persist after withdrawal or if its removal amplifies pain. To explore this, we extended our previous sample^17^ to ensure sufficient power to detect order effects.

## 2. Methods

### 2.1. Participants

Sixty-five couples participated: 37 (age: mean [SD] = 21.5 [3.8]) were recruited for,^17^ and 28 were added (age: mean [SD] = 20.4 [2.75]) to achieve adequate power for the analysis of the effect of condition order. The mean (SD) age of the full sample was 21 (3.3) years. To avoid the confounding effects of sex, we always had the woman undergo the electrical stimulation, inducing central sensitization. Indeed, as women and men have (1) a different threshold for pain and (2) potential differences in the development of secondary mechanical hypersensitivity.^4,6,11,15,19^ Exclusion and inclusion criteria followed our previous criteria^17,18^ and are detailed in the supplementary material, http://links.lww.com/PR9/A417. The study was approved by the Social and Societal Ethics Committee (SMEC) of KU Leuven (G-2020-2854). Here we use the more precise term mechanical hypersensitivity instead of mechanical hyperalgesia, as used in the original publication^17^, to align with the IASP definition, but it refers to the same measure. So, in this context, the 2 terms should be seen as interchangeable.

### 2.2. Sample size estimation

We added participants to an existing sample of 37,^17^ based on a power analysis to detect order effects.^33,34^ A Bayesian stopping rule guided recruitment: after every 5 new participants, we evaluated the Bayes Factor (BF_10_) for the order effect. Data collection was terminated when one of 3 prespecified criteria was met: (1) the Bayes factor (BF_10_) reached 10, indicating strong evidence for either the null or the alternative hypothesis; (2) a maximum of 40 additional participants had been recruited; or (3) resource limitations were reached. In practice, data collection was stopped after 28 additional couples because of time and resource constraints, and because the Bayes factor showed no meaningful change with further increases in sample size.

### 2.3. Induction of secondary mechanical hypersensitivity: middle frequency stimulation

**Figure 1.**
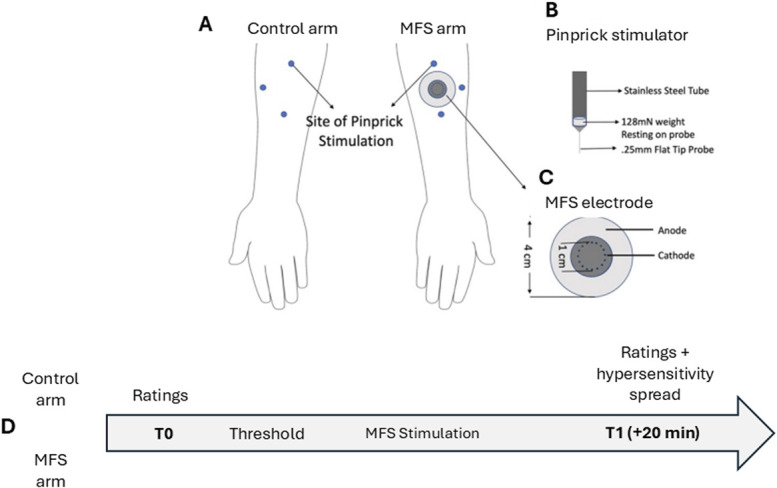
Experimental setup and procedure (adapted from ^17^). (A) Blue dots represent the site of pinprick stimulation, and the site of the electrode on the arm represents the location of the MFS stimulation. Three pinpricks were delivered to each arm at each time point at the locations corresponding to the 3 blue dots on each arm. (B) Details of the handheld pinprick stimulator. (C) Details of the electrode used for MFS. (D) Timeline of the experiment. Pinprick sensitivity was assessed at T0 (before) and T1 (20 minutes) poststimulation. After T0, the MFS stimulation threshold was established. The participant and their partner held hands only during the 2-minute MFS stimulation. After a 20-minute wait, the areas of mechanical hypersensitivity and pinprick sensitivity were measured at T1. The whole experiment lasted approximately 60 minutes. MFS, middle frequency stimulation.

Middle frequency stimulation of the volar forearm skin consisted of 12 trains of electrical pulses (each train lasted 1 second of 2-ms pulses at 42 Hz) delivered over 2 minutes.^8,42^ The intertrial interval was 9 seconds. The stimuli were delivered using a custom-built electrode^40,41^ consisting of a cathode with 16 blunt stainless-steel pins, each 0.2 mm in diameter, protruding 1 mm from the base and arranged in a circle with a 10 mm diameter. The anode consisted of a surrounding stainless-steel ring with an inner diameter of 22 mm and an outer diameter of 40 mm (Fig. 1). The electrode was controlled via a DS5 Isolated Bipolar Constant Current Stimulator (Digitimer, Welwyn Garden City, United Kingdom). Participants rated the intensity and unpleasantness of each train on visual NRS scales (0-100). For intensity, 0 = no sensation, 50 = pain threshold, and 100 = most intense pain imaginable. For unpleasantness, 0 = not unpleasant and 100 = most unpleasant imaginable.

### 2.4. Mechanical pinprick stimulation

A calibrated 128-mN handheld pinprick stimulator with a cylindrical stainless steel 0.25-mm diameter flat-tip probe was used (MRC Systems, Heidelberg, Germany). The tube was held perpendicular to the skin by the experimenter and moved down and up with a total duration of ∼1 second. The location of the pinprick stimulus was displaced after each pinprick. Three pinpricks were applied to each arm at each time point. The arm onto which pinpricks were first applied (dominant vs nondominant) was randomized.

### 2.5. Measurements

*Pinprick ratings* for perceived intensity were collected for each pinprick stimulus on a scale of 0 to 100, where 0 was no sensation at all, 50 marked the transition from nonpainful to painful, and 100 represented the most intense pain imaginable. Unpleasantness ratings were also collected for each of the 3 stimuli on a scale from 0 to 100 where 0 represented not unpleasant at all and 100 represented extremely unpleasant.

*The spread of secondary mechanical hypersensitivity* (on the middle frequency stimulation [MFS] arm) was quantified using the 128-mN pinprick stimulator, by stimulating along 4 linear paths arranged vertically (rostral–caudal) and horizontally (lateral–medial) along the stimulated forearm in ∼1-cm steps. The stimulation started at the wrist and at the cubital fossa for the vertical axis and at the inner and outer edges of the forearm for the horizontal axis and moved towards the center of the stimulation site until the participant reported a clear change in pinprick intensity. The point at which the change occurred was then marked. The total length was then measured along both the vertical (length) and horizontal (width) axes. See Figure 1 for the full experimental procedure.

Participants also reported their *levels of support and stress* during MFS on scales ranging from 0 (not supported at all, not stressed at all) to 10 (completely supported, most stressed imaginable).

### 2.6. Procedure

We followed the procedure described in ^17^ focusing only on behavioral outcomes (see ^17^ and Supplementary Material, http://links.lww.com/PR9/A417). Participants attended 2 sessions, 1 week apart, receiving MFS either alone or while their partner held their hands. Condition order was randomly assigned. On arrival, the participant and partner sat facing each other while the experimenter explained the protocol and obtained consent. Baseline ratings of pinprick intensity and unpleasantness on both arms were collected (T0; Fig. 1). In the alone condition, the partner was escorted to another room; in the handholding condition, both remained seated together. The stimulation electrode was placed pseudorandomly on the dominant or nondominant forearm, midway between wrist and cubital fossa (Fig. 1). Detection threshold was determined with a staircase procedure Before MFS, partners received written instructions on providing support through handholding (see Ref.^17^ and Supplementary Material, http://links.lww.com/PR9/A417). MFS was calibrated to 10× detection threshold, and participants rated the intensity and unpleasantness of each train. After MFS, participants rated fear, perceived support, and stress (0–10 NRS). After a 20-minute break without emotionally arousing content, pinprick ratings were repeated on both arms (T1), and vertical and horizontal hypersensitivity spread was measured on the MFS arm. Participants were then debriefed and compensated. Each session lasted about 60 minutes.

### 2.7. Statistical analysis

All statistical analyses were conducted in R.^29^ To account for the clustered nature of the repeated measures data, we fit linear mixed models using the lmer function from the lme4 package,^3^ with a random intercept for each subject. Significant interactions were followed up using either the emmeans^23^ or simple slopes^16^ functions, as appropriate, with Tukey multiple comparison correction. Effect sizes are reported throughout, with follow-up contrasts quantified using model-based standardized effect sizes that appropriately reflect the hierarchical structure of the data. Bayes factors were computed, as a complementary inferential measure, to quantify the evidence in favour of the alternative hypothesis. Default uninformative priors (Cauchy distribution; scale = 0.707) were used. Analyses followed the preregistered plan (https://doi.org/10.17605/OSF.IO/7BXPG).

### 2.8. Perceived support, stress, and middle frequency stimulation ratings

Ratings of support and perceived stress were analysed using 2-tailed paired sample *t* tests. The ratings for the MFS stimulation were analyzed using linear mixed-effects models (separately for intensity and unpleasantness), with condition and order as fixed factors and participant as a random grouping factor. The ratings for each of the 12 trains were entered into the model.

### 2.9. Primary outcomes

#### 2.9.1. Pinprick ratings

Separate models were fitted for pinprick intensity, pinprick unpleasantness, and spread of mechanical hypersensitivity. All 3 pinprick ratings were included in the model, with baseline ratings (T0) modelled as a covariate. Condition (handholding vs alone) and arm (control vs MFS; pinprick ratings only) were included as fixed factors, with a random intercept for participant. The order of condition presentation was then added to the model as an additional fixed factor (0 = alone first; 1 = support first). Our primary interest was the 3-way interaction between order, condition, and arm, to test whether the order of presentation influenced the condition × arm effect. We also computed a Bayes factor (BF10) for this three-way interaction by comparing the full model with a null model excluding the interaction. The stopping rule described above was based on this Bayes factor.

#### 2.9.2. Spread of mechanical hypersensitivity

A similar approach was followed to analyse the vertical and horizontal spread of mechanical hypersensitivity. The model fitted was: spread ∼ condition × order + (1|participant). We expected a significant main effect of condition and a significant interaction between condition and order. In addition, we computed a Bayes factor (BF10) to quantify the evidence in favour of the alternative hypothesis (the condition × order interaction).

## 3. Results

Data collection was stopped after 28 couples due to time and resource constraints, and because there was no further appreciable change in the Bayes factor. The final sample comprised 65 couples. Only the results of the main hypotheses of interest (relevant main effects and interactions) are reported in the text below. The full model outputs are presented in the supplementary material (Tables S2–S16, http://links.lww.com/PR9/A417).

### 3.1. Perceived support, stress, and middle frequency stimulation ratings

**Figure 2.**
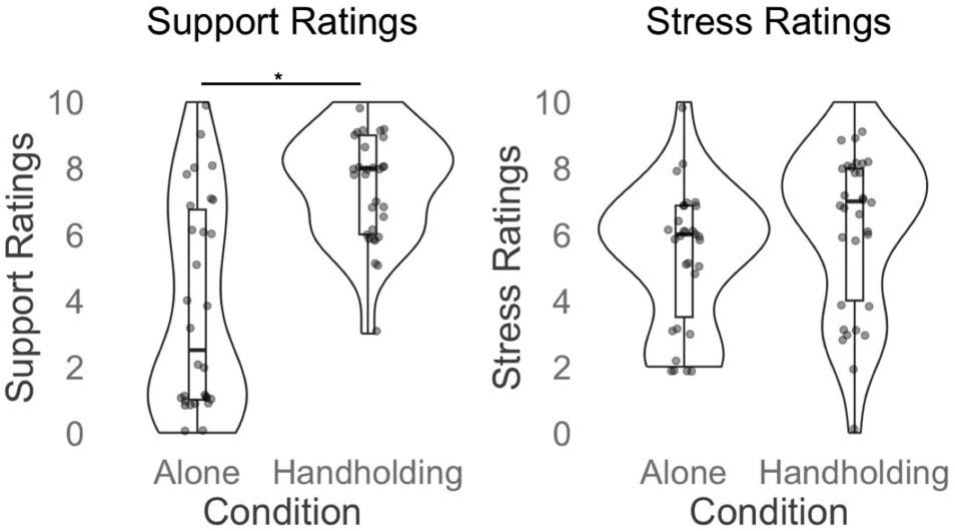
Perceived support and stress ratings. (A) Perceived support ratings (B) Perceived Stress ratings. The perceived support ratings were significantly higher in the support condition relative to the alone condition. However, the perceived stress ratings did not differ significantly between the 2 conditions. Boxplots represent the interquartile range of the distributions, and the violin plots represent the kernel density. Asterisks indicate statistical significance at *P* < 0.05.

Our manipulation was successful, as indicated by significantly higher ratings of perceived support in the handholding condition compared with the alone condition (t [60] = 12.98, *P* < 0.001; mean [SD]: support = 7.69 [1.46], alone = 3.39 [2.89]; Cohen d = 1.66; BF_10_ > 10). In contrast, stress ratings did not significantly differ between the 2 conditions (t [60] = 0.27, *P* = 0.78; mean [SD]: support = 5.86 [2.44], alone = 5.73 [2.47]; Cohen d = 0.04; BF_10_ = 0.14) (Fig. 2).

### 3.2. Middle frequency stimulation intensity ratings

**Figure 3.**
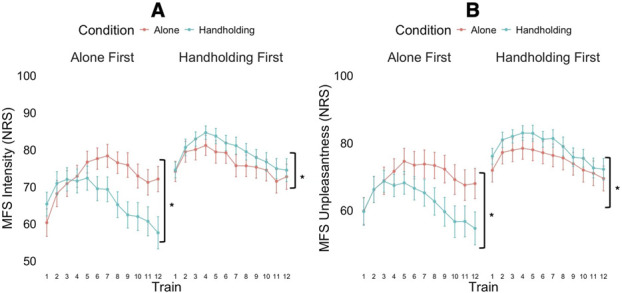
Perceived intensity (A) and unpleasantness (B) of MFS stimulation. The plots represent the mean perceived intensity and unpleasantness of the MFS stimulation. The dots and error bars represent the mean across participants and the respective standard errors for each train of MFS stimulation. The asterisk in both plots signifies a significant difference between the 2 order groups across all trains of stimulation (average rating per participant). The average rating in the support condition was significantly lower than that in the alone condition for both order conditions, but this difference was smaller in the support first group, owing to higher ratings in the support condition when it was experienced first. Asterisks indicate statistical significance at *P* < 0.05. MFS, middle frequency stimulation; NRS, numerical rating scale.

Middle frequency stimulation intensity ratings showed a significant condition × order interaction (F [1, 1409.97] = 55.34, *P* < 0.001, $\eta_{p}^{2}$ = 0.038). In the alone condition, ratings did not differ by order (alone first: M = 73.40, SD = 16.07; alone second: M = 76.55, SD = 14.87; b = −3.71, *P* = 0.28, d = −0.34). However, in the handholding condition, ratings were significantly higher when handholding was experienced first (M = 79.96, SD = 10.91) vs second (M = 66.56, SD = 16.57; b = −12.01, *P* = 0.001, d = −1.12) (Figs. 3A and B). Middle frequency stimulation unpleasantness ratings showed a similar pattern (Table 1 and Fig. 2, see supplementary material, http://links.lww.com/PR9/A417 for the unpleasantness results, which overlapped with the intensity results).

**Table 1:** Middle frequency stimulation ratings.

	Alone M (SD)	Support M (SD)
MFS intensity ratings		
Alone first	73.40 (16.08)	66.57 (16.58)
Support first	76.56 (14.88)	79.96 (10.91)
MFS unpleasantness ratings		
Alone first	70.75 (21.04)	62.67 (19.36)
Support first	74.88 (18.08)	79.11 (12.41)

Mean and standard deviation of the perceived intensity (upper panel) and unpleasantness (lower panel) ratings in response to the MFS stimulation. The analyses of the unpleasantness ratings are presented in the supplementary material, http://links.lww.com/PR9/A417.

MFS, middle frequency stimulation.

### 3.3. Primary outcomes

#### 3.3.1. Pinprick stimulation perceived intensity

**Figure 4.**
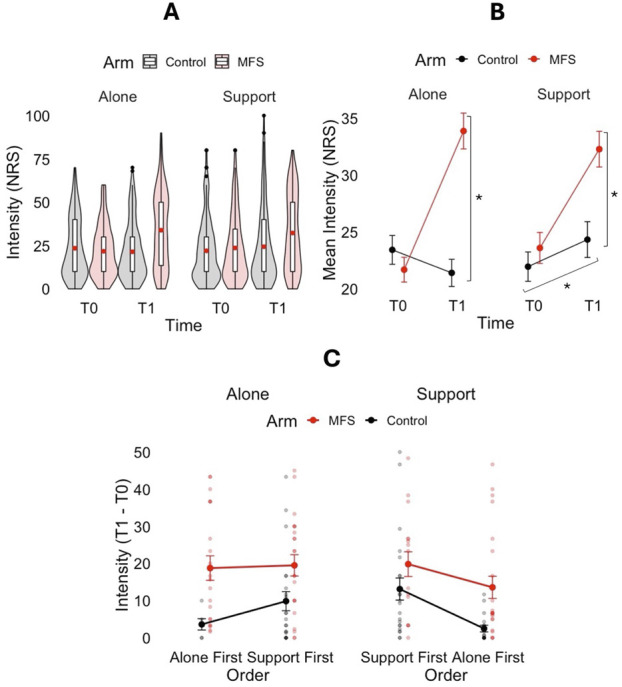
Pinprick intensity ratings. (**A**) Raw pinprick intensity ratings (NRS) in response to pinprick stimulation, split by *arm* (MFS vs control), *time* (T0 vs T1), and *condition* (alone vs support). Red dots represent the group mean; boxplots show the interquartile range and median, and violins illustrate the distribution density. (B) Model-predicted mean intensity ratings with standard errors, plotted by *time*, *arm*, and *condition*. Panel (B) depicts the same information as in panel (A) but is represented in a way that better captures the interaction effect, particularly the change in ratings over time on the control arm. (C) Change in intensity ratings (T1 − T0), shown by *order* group (alone first vs support first), *condition*, and *arm*. Dots represent individual participants; lines and error bars reflect group means and standard errors. Asterisks indicate statistical significance at *P* < 0.05. MFS, middle frequency stimulation; NRS, numerical rating scale.

We observed a significant condition × arm interaction (F [1,654.23] = 7.41, *P* = 0.007, $\eta_{p}^{2}$ = 0.011), indicating that handholding influenced secondary mechanical hypersensitivity. Although hypersensitivity was present in both conditions, the arm difference was greater in the alone condition (b = −12.28, SE = 1.36, *P* < 0.001, d = −1.14) than in the support condition (b = −7.02, SE = 1.37, *P* < 0.001, d = −0.74). Follow-up comparisons showed higher ratings on the control arm in the support condition (M = 24.90, SD = 21.74) than in the alone condition (M = 22.03, SD = 16.36; b = −2.87, SE = 1.37, *P* = 0.03, d = −0.21), whereas ratings on the MFS arm did not differ significantly (b = 2.39, SE = 1.37, *P* = 0.08, d = 0.18). This suggests that the interaction may be driven by reduced habituation on the control arm in the support condition. To test whether the condition × arm interaction was driven by reduced habituation on the control arm in the support condition, we ran a model with time, arm, and condition as fixed factors. This revealed a significant time × arm × condition interaction (F [1,1415.5] = 7.76, *P* = 0.005, $\eta_{p}^{2}$ = 0.005). In the alone condition, control arm ratings did not change from T0 to T1 (b = 1.16, SE = 1.29, *P* = 0.36, d = 0.09), whereas in the support condition, they increased significantly (b = −2.92, SE = 1.30, *P* = 0.024, d = −0.23). On the MFS arm, ratings increased in both conditions, but more so in the alone condition (b = −11.63, SE = 1.29, *P* < 0.001, d = −0.93) than in the support condition (b = −8.51, SE = 1.30, *P* < 0.001, d = −0.68) (Fig. 4; Table 2).

**Table 2:** Pinprick ratings**.**

Pinprick intensity ratings								
Order	Alone				Support			
	Control		MFS		Control		MFS	
	T0	T1	T0	T1	T0	T1	T0	T1
Alone first	23.25 (16.68)	16.77 (13.77)	21.32 (13.53)	32.18 (23.2)	14.22 (12.29)	13.57 (12.9)	14.98 (13.35)	26.51 (21.1)
Support first	23.58 (16.86)	26.98 (17.24)	23.53 (16.85)	35.42 (19.36)	29.84 (18.63)	35.18 (23.14)	32.52 (19.1)	37.4 (19.98)

Pinprick unpleasantness ratings								
Order	Alone				Support			
	Control		MFS		Control		MFS	
	T0	T1	T0	T1	T0	T1	T0	T1
Alone first	15.06 (15.89)	11.11 (12.48)	14.51 (12.31)	26.33 (22.84)	9.3 (10.41)	9.26 (12)	9.16 (9.79)	25.93 (23.23)
Support first	17.47 (15.95)	20.38 (17.99)	19.21 (17.83)	27.34 (19.44)	24.71 (19.57)	27.79 (23.85)	26.64 (21.16)	28.68 (20.76)

Mean and standard deviation of the perceived intensity (upper panel) and unpleasantness (lower panel) ratings in response to the mechanical pinprick stimulation.

MFS, middle frequency stimulation.

Our main target of interest, the three-way condition × arm × order interaction (when controlling for the ratings at T0), was not significant (F [1,652.53] = 1.04, *P* = 0.30, $\eta_{p}^{2}$ = 0.001), suggesting that the order in which participants experienced the 2 conditions did not significantly influence the impact of handholding on secondary mechanical hypersensitivity. However, Bayesian analysis provided strong evidence in favour of the model including the three-way interaction over a model without it, with a Bayes factor (BF10) of 12,844.68 (Fig. 4).

#### 3.3.2. Pinprick stimulation perceived unpleasantness

**Figure 5.**
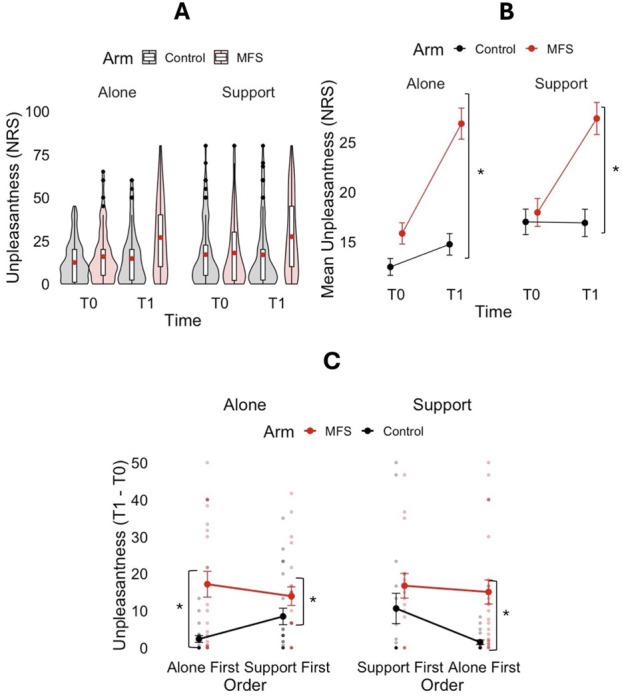
Pinprick unpleasantness ratings. (A) Raw pinprick unpleasantness ratings (NRS) in response to pinprick stimulation, split by *arm* (MFS vs control), *time* (T0 vs T1), and *condition* (alone vs support). Red dots represent the group mean; boxplots show the interquartile range and median, and violins illustrate the distribution density. (B) Model-predicted mean unpleasantness ratings with standard errors, plotted by *time*, *arm*, and *condition*. This panel summarizes the same data as panel (A) but highlights the interaction effects more clearly. (C) Change in unpleasantness ratings (T1 − T0), shown by *order* group (alone first vs support first), *condition*, and *arm*. Dots represent individual participants; lines and error bars reflect group means and standard errors. Asterisks indicate statistical significance at *P* < 0.05. MFS, middle frequency stimulation; NRS, numerical rating scale.

Consistent with,^31^ there was no significant arm × condition interaction for pinprick unpleasantness ratings (F [1,649.89] = 1.54, *P* = 0.21, $\eta_{p}^{2}$ = 0.002). However, a significant condition × arm × order interaction emerged (F [1,649.86] = 3.88, *P* = 0.049, $\eta_{p}^{2}$ = 0.006; Fig. 5). Separate analyses showed significant arm × order interactions in both the support (F [1,268.84] = 30.55, *P* < 0.001, $\eta_{p}^{2}$ = 0.102) and alone (F [1,286.45] = 15.67, *P* < 0.001, $\eta_{p}^{2}$ = 0.052) conditions. In the alone condition, hypersensitivity was present regardless of order but was reduced when preceded by support (b = −6.47 vs b = −15.34), compared with when it was experienced first (b = −15.34, SE = 1.61, *P* < 0.001, d = 1.42). In the handholding condition, hypersensitivity (main effect of arm) emerged when participants experienced the stimulation after the alone condition (alone first) (b = −16.72, SE = 2.19, *P* < 0.0001, d = −1.15) whereas there was no significant effect of arm (no secondary mechanical hypersensitivity) when the handholding condition was experienced first (b = 0.04, SE = 2.09 *P* = 0.98, d = 0.002) (Fig. 5). Further follow-ups revealed that ratings on the MFS arm did not differ between the order groups in either the alone condition (b = 0.00, SE = 3.91, *P* = 1, d = 0.05) or the support condition (b = −0.11, SE = 4.02, *P* = 1, d = 0.31). For the control arm, there was no significant difference in ratings between the order groups in the alone condition (b = −8.64, SE = 3.90, *P* = 0.12, d = 0.05). However, in the support condition, ratings were significantly higher when the support condition was experienced first compared with when it was experienced second (alone first) (b = 16.32, SE = 3.99, *P* < 0.001, d = 0.8). See Table 2 (bottom panel) for descriptive statistics.

Bayesian analysis provided strong evidence in favour of the model including the three-way interaction with order over a model without it, with a Bayes Factor (BF10) of 31,253,171.

#### 3.3.3. Vertical spread

**Figure 6.**
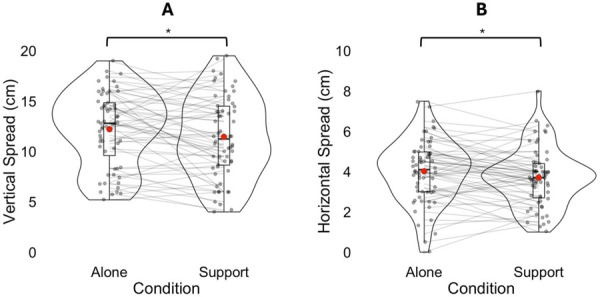
Vertical and horizontal spread of secondary mechanical hypersensitivity. (**A)** Vertical spread and (**B)** horizontal spread split by condition: *alone* and *support*. Individual data points are overlaid in grey, boxplots represent the interquartile range and median, violins represent the kernel density of the distribution, and red dots indicate the group mean. Asterisks indicate statistical significance at *P* < 0.05.

The vertical spread of mechanical hypersensitivity was significantly larger in the alone (M = 12.2, SE = 0.53) than handholding (M = 11.2, SE = 0.53) condition (main effect of condition) (F [1,58.87] = 7.49, *P* = 0.008, $\eta_{p}^{2}$ = 0.113), alone vs handholding (b = 1.09, SE = 0.37, *P* = 0.0082, d = 0.49) (Fig. 6). In this case, the order did not have any effects, either as main effects (F [1,59.64] = 1.06, *P* = 0.307, $\eta_{p}^{2}$ = 0.02), or in interaction with the condition (condition × order interaction) (F [1,58.87] = 0.017, *P* = 0.896, $\eta_{p}^{2}$ < 0.001). Bayesian analysis yielded a Bayes factor for the alternative hypothesis (BF10) of 0.27, providing weak evidence in favour of the model without the interaction with order.

#### 3.3.4. Horizontal spread

We replicated our previous finding that handholding affects the horizontal spread of mechanical hypersensitivity (F [1,59.24] = 5.29, *P* = 0.025, $\eta_{p}^{2}$ = 0.082). The horizonal spread was larger in the alone (M = 4.02, SE = 0.19) than in the handholding condition (M = 3.68, SE = 0.19, [b = 0.34, SE = 0.15, *P* = 0.025, d = 0.41]) (Fig. 6). The order had no statistically significant effect (the main effect of order) (F [1,59.95] = 0.0007, *P* = 0.979, $\eta_{p}^{2}$ = < 0.001); condition × order interaction (F [1,59.24] = 0.96, *P* = 0.330, $\eta_{p}^{2}$ = 0.02). Bayesian analysis provided weak evidence against the inclusion of the condition × order interaction, with a Bayes factor (BF10) of 0.40.

### 3.4. Exploratory analyses

#### 3.4.1. Effect of order and condition on baseline pinprick ratings at T0

**Figure 7.**
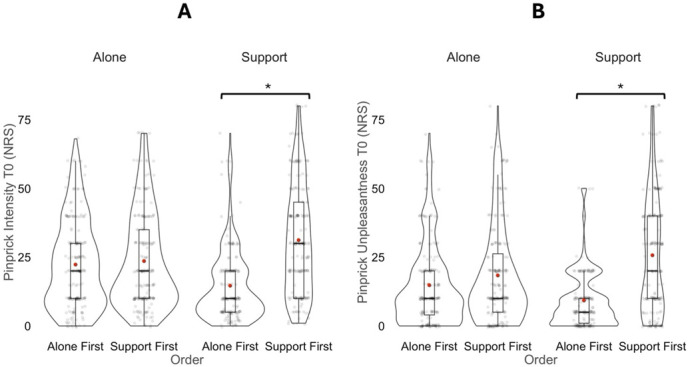
Pinprick intensity and unpleasantness ratings at T0 (baseline): (A) violin plots showing baseline pinprick intensity ratings (T0) on a numerical rating scale (NRS), split by *condition* (alone, support) and *order* group (alone first, support first). (B) Violin plots showing baseline pinprick unpleasantness ratings (T0), plotted using the same grouping. Red dots indicate group means; boxplots represent the interquartile range and median, and violins indicate the kernel density of the distribution. Asterisks indicate statistical significance at *P* < 0.05.

To explore how order affected baseline pinprick sensitivity, we analyzed T0 ratings (intensity and unpleasantness) with fixed effects for condition (alone vs handholding), arm (MFS vs control), and order (alone first vs handholding first). For intensity ratings, we found a significant condition × order interaction (F [1, 682.10] = 96.81, *P* < 0.001, $\eta_{p}^{2}$ = 0.124). In the alone condition, baseline ratings did not differ between order groups (alone first: M = 22.28, SD = 16.20; handholding first: M = 23.56, SD = 17.44; b = −2.11, *P* = 0.571, d = −0.21). However, in the support condition, ratings were significantly higher when handholding came first (M = 31.18, SD = 19.90) vs second (M = 14.60, SD = 13.13; b = −16.14, *P* < 0.001, d = −1.67) (Fig. 7). Unpleasantness ratings showed a similar pattern with a significant condition × order interaction (F [1, 681.69] = 84.93, *P* < 0.001, $\eta_{p}^{2}$ = 0.111). The alone condition showed no order difference (alone first: M = 14.78, SD = 15.10; handholding first: M = 18.34, SD = 17.72; b = −3.98, *P* = 0.296, d = −0.46), whereas the support condition showed significantly higher ratings when experienced first (M = 25.67, SD = 21.33) vs second (M = 9.23, SD = 10.30; b = −15.75, *P* < 0.001, d = −1.8) (Fig. 7, Table 3). Further analyses conducted on the 2 samples separately confirmed the consistency of these findings and are reported in the supplementary material, http://links.lww.com/PR9/A417, along with another confirmatory analysis presenting the results split per order of effect and session number. This latter analysis aimed to provide conceptual clarity, even if it was statistically redundant.

**Table 3:** Mean and standard deviation of the perceived intensity (upper panel) and unpleasantness (lower panel) ratings in response to the mechanical pinprick stimulation at T0, split by condition order (alone first vs support first) and session number (first vs second).

Baseline pinprick intensity ratings		
	First session	Second session
Alone first	22.3 (16.2)	14.6 (13.1)
Support first	31.2 (19.9)	23.6 (17.4)

Baseline pinprick unpleasantness ratings		
	First session	Second session
Alone first	14.8 (15.1)	9.23 (10.3)
Support first	25.7 (21.3)	18.3 (17.7)

## 4. Discussion

In the present study, handholding (and staying with the partner after electrical stimulation) reduced both the vertical and horizontal spread of mechanical hypersensitivity, regardless of order^17^ It also reduced differences in perceived pinprick intensity, although this effect seemed driven by higher control-arm scores at T1 and was not observed for unpleasantness ratings or in earlier social support studies.^25,30^ Effects on pinprick ratings depended on condition order through baseline sensitivity, with higher baseline ratings when handholding was administered first. Post-MFS ratings, however, did not differ by condition or order, suggesting an inverse relationship between baseline sensitivity and post-MFS increases. The findings on hypersensitivity spread strengthen our previous report^17^ particularly because vertical spread shows greater between-session reliability than horizontal spread.^8^ These effects are unlikely to be explained by perceived stress, as stress ratings did not differ between conditions, in line with our prior findings showing no effect of handholding on stress physiology.^17,18^

The effect of condition order on pinprick ratings appeared driven by baseline sensitivity, which was highest when handholding came first and lowest when it followed the alone condition (see exploratory analyses). This pattern was consistent across both subsamples and across MFS intensity and unpleasantness ratings, suggesting that handholding's impact depends on when it is received. Although solicitous support can increase pain^12,46^this is unlikely here given the controlled intervention. Instead, 2 behavioral explanations may apply. The intimacy model^7^ suggests that verbal pain ratings enhance intimacy in close relationships, whereas the communal coping model proposes that pain expression signals need and elicits support.^35,36^ When support is available during initial threat exposure, it may facilitate this signaling to strengthen intimacy or recruit support. In addition, lower ratings of mechanical sensitivity when handholding followed the alone condition are consistent with the safety signaling hypothesis^20,21,45^ which proposes that support reduces the salience of noxious input. This suggests that support may signal safety after a potentially threatening situation. The effect may be amplified by contrast or relief, as receiving support after its absence may induce relief^22^ or reduce stress. However, this interpretation should be treated cautiously, as stress ratings did not differ significantly between conditions, regardless of order.

Elevated baseline ratings in the support-first condition persisted into the alone condition, reducing the arm effect. This pattern, seen for both MFS intensity and unpleasantness, may reflect frustrative nonreward (FNR), ^1,2,28,48^ in which withdrawal of expected support heightens arousal and negative affect,^26,27,28^ potentially increasing pain sensitivity or disrupting habituation.Unlike simple nonreward, FNR involves violated expectations,^27^ participants may have expected continued support, and its absence may have disrupted habituation. This is consistent with placebo/nocebo research showing that expectancy violation can abolish analgesia.^9^

From a habituation perspective (see supplementary analysis, http://links.lww.com/PR9/A417), ratings were lower in the second session regardless of handholding, and overall higher when handholding was experienced first. The overall reduction likely reflects habituation. The greater reduction when handholding followed the alone condition may indicate amplified or additive effects of handholding on habituation. In contrast, the smaller reduction when handholding preceded the alone condition may reflect anchoring effects or the negative impact of losing support. The spread of hypersensitivity showed no evidence of habituation: both vertical and horizontal spread were consistently smaller in the support condition, independent of order. Notably, the baseline and control-arm effects described above fully accounted for all significant differences in subjective ratings. Poststimulation increases on the stimulated arm varied inversely with baseline ratings, consistent with the law of initial value,^47^ which holds that response magnitude depends on prestimulus level. Although several studies have used similar electrical stimulation model to examine mechanical hypersensitivity^18^,^21^,^22^,^28^,^41^,^43^ such systematic baseline differences have not previously been reported.

In summary, handholding robustly reduced the spread of mechanical hypersensitivity, regardless of habituation or order, whereas its effects on the perception of mechanical stimuli before and after central pain plasticity were more complex and timing-dependent. Clinically, this suggests that social support may have relatively stable effects on spinal nociceptive processes, whereas perception of mechanical stimuli is more sensitive to context. Future studies should examine how the sequence of supportive interactions influences pain and mechanical hypersensitivity in men, and how support or its absence shapes these processes over longer time frames and across changing contexts.

## Disclosures

The authors have no conflict of interest to declare.

## Supplemental digital content

Supplemental digital content associated with this article can be found online at http://links.lww.com/PR9/A417.
