# Supplementary material for: Do partner handholding effects linger? Order effects of handholding support on mechanical hypersensitivity in women
Source: Pain Rep. 2026 Jul 17;11(4):e1464. doi: 10.1097/PR9.0000000000001464 (PMC13384690; doi:10.1097/PR9.0000000000001464)
Supplement: Supplementary file 1 [file painreports-11-e1464-s001.pdf]

Supplementary Material

Supplementary methods

Inclusion criteria

Participants were excluded from the study if they met any of the following exclusion criteria: a history of chronic pain or pain on the day of the experiment, a diagnosis of depression, anxiety, substance use disorder, or other psychiatric conditions, the presence of injuries or scars on the forearm, regular use of prescribed or non-prescribed psychotropic medication, pregnancy, or insufficient sleep (<5 hours) the night before the experiment. Additionally, participants were required to abstain from consuming any analgesics, anti-inflammatory medications, or stimulants such as nicotine or caffeine for at least 12 hours before the start of the experiment. Eligibility was further restricted to individuals who had been in a romantic relationship for at least one year. While the study did not explicitly limit recruitment to heterosexual couples, all participants who volunteered were in heterosexual relationships. These exclusion and inclusion criteria were verified based on the participants' self-reports. Participants were recruited through the online Experiment Management System (EMS) of the Faculty of Psychology and Educational Sciences at KU Leuven. Before participation, all individuals provided written informed consent. Both participants and their partners received compensation in the form of course credits or financial reimbursement (1 credit/hour or €10/hour).

Extended procedure (adapted from Jaltare et al., 2023)

All participants attended two experimental sessions (Alone and Handholding conditions) separated by 1 week. The order of the two experimental conditions was counterbalanced across participants. Upon the couple's arrival at the laboratory, the participant and her partner were invited to sit at a table facing each other. The experimenter explained the study protocol, and the participant was invited to read and sign the informed consent form. Afterwards, the experimenter collected the participant's

baseline ratings of intensity and unpleasantness for the mechanical pinprick stimuli that were applied on both arms (T0, see following paragraphs for details).

In the *Alone condition*, the partner was then invited to sit in an adjacent room. The participant did not have any prior knowledge that the partner would be asked to leave the room in the alone condition. In the *Handholding condition*, the participant and her partner remained in the same room. The stimulation electrode was placed on the participant's volar forearm. The arm onto which the electrode was placed (dominant vs non-dominant) was randomized across participants. The participant's detection threshold was then established using a staircase procedure where a low intensity was presented at first, and then was gradually increased (.1mV step size) until the participant was able to detect the stimulus. The intensity was then lowered again until the participant was unable to detect the stimulus. The threshold was established after three such reversals. In the support condition, before performing the MFS stimulation on the participant, the partner received the following written explanation in English and Dutch: *"We will now proceed with the stimulation. Your role here is to provide support to your partner. During the stimulation procedure, you will be required to hold both your partner's hands in any way that is most comfortable to you and your partner or in any way that you think will be most supportive to your partner."* There were no specific instructions given to the partner regarding supporting the participant with verbal support, however, no verbal support was provided by any of the participant's partners. The experimenter informed the participant that her partner would be holding her hands only during the stimulation, and MFS was delivered after handholding started. The intensity of MFS stimulation was calibrated at 10x the participant's detection threshold. Participants provided a rating for the intensity and unpleasantness for each MFS train. The MFS electrode was subsequently removed. Participants then rated their fear of MFS, and perceived support and stress during MFS on a Numeric Rating Scale (NRS) between 0 and 10. Participants were then asked to wait for a period of 20 mins before pinprick ratings would be collected again at T1. During the waiting period participants were left free to interact in the support condition but were

instructed not to discuss topics that were emotionally arousing, and/or watch emotionally arousing content on their phone. In the alone condition the participant was asked to refrain from watching arousing content on their phone.

Twenty (T1) minutes after the end of MFS, mechanical pinprick intensity and unpleasantness ratings were collected on both the MFS and control arm. The proximal-distal length and the lateral-medial width of increased sensitivity to pinprick stimuli were also measured on the MFS arm at T1. At the end of the second testing session, each couple was debriefed and compensated.

Instructions to the partner for the support

We will now proceed with the stimulation. Your role here is to provide support to your partner. During the stimulation procedure, you will be required to hold both your partner's hands in any way that is most comfortable to you and your partner or in any way that you think will be most supportive to your partner.

Questionnaire

Adult Attachment Styles: Adult attachment styles were measured using the Experiences in Close Relationships - Revised questionnaire. The ECR-R is a 36-item questionnaire that yields attachment scores on two dimensions: attachment anxiety and avoidance. Scores on both dimensions range from 1 (low) to 7 (high). Lower scores indicate greater attachment security, and higher scores indicate attachment insecurity. Attachment styles were measured to follow the methodology of [1]. However, given that the primary aim of this study was to explore the role of the order in which participants experienced the two conditions, attachment styles have not been incorporated into the analysis as the current sample size was not adequately powered to detect an interaction with attachment styles.

Supplementary results

MFS Unpleasantness Ratings

We also found a significant interaction between condition and order for the MFS unpleasantness ratings ($F(1, 1409.65) = 62.66$, $p < .001$, $\eta p^2 = 0.043$). Follow-ups revealed that in the alone condition, there was no significant difference between the alone first and support first groups ($b = -5.82$, $SE = 4.03$, $p = 0.15$, $d = .40$). The mean ratings when participants experienced the alone condition first were marginally lower (M (SD) = 70.75 (21.03)) than those in the support first group (M(SD) = 74.88 (18.07)). For the support condition, the MFS ratings in the support first group were significantly higher (M(SD) = 79.10 (12.41)) compared to the alone first group (where the support condition was experienced second) (M = 62.67 (19.35)) ($b = -12.01$, $SE = 3.47$, $p = .001$, $d = -1.2$). These results were consistent with those of the MFS intensity ratings. See Table 1 for descriptive statistics.

Exploratory analysis of the combined effect of Order and Session Number

Pinprick Intensity T0: An exploratory analysis examining habituation effects across sessions (a factor we called number) revealed significant main effects of number ($F(1, 686.11) = 96.54$, $p < .001$, $\eta p^2 = 0.123$) and order ($F(1, 61.86) = 6.30$, $p = .015$, $\eta p^2 = 0.092$), but no number × order interaction ($F(1, 686.11) = 0.11$, $p = .746$, $\eta p^2 < 0.001$). The support first group showed higher baseline ratings (M = 27.42, SD = 19.09) than the alone first group (M = 18.56, SD = 15.26; $b = -9.12$, $p = .015$, $d = -.94$). Ratings were also higher in session one (M = 26.86, SD = 18.71) than session two (M = 19.29, SD = 16.15; $b = 7.01$, $p < .001$, $d = .72$) across both groups (Figure 8A).

Pinprick Unpleasantness T0: Similarly, for the pinprick unpleasantness ratings, we found significant main effects of order ($F(1, 61.95) = 7.03$, $p = .010$, $\eta p^2 = 0.102$) and number ($F(1, 685.70) = 84.86$, $p < .001$, $\eta p^2 = 0.110$), but no significant order x number interaction ($F(1, 685.70) = 2.57$, $p = .109$, $\eta p^2 = 0.004$). Participants in the support first group reported significantly higher baseline pinprick unpleasantness ratings (M = 22.06, SD = 19.95) compared to those in the alone first group (M = 12.1, SD = 13.27; $b = -9.86$, $SE = 3.72$, $p = .010$, $d = -1.14$). Across both orders, unpleasantness ratings were significantly higher in the first session (M = 20.39, SD = 19.33) than in the second session (M = 14.00,

SD = 15.34; b = 5.89, SE = 0.64, p < .001, d = .68) (Figure 8B). The means and SDs split by order and session number are presented in Table 4 below.

Vertical Spread: For the vertical spread of mechanical hypersensitivity, we found a significant number x order interaction (F(1, 58.87) = 7.49, p = .008, ηp^2 = 0.113). Follow-ups of the interaction revealed that in the alone first group, there was no significant difference in spread between the first (M = 12.7, SD = 4.05) and second sessions (M = 11.9, SD = 4.30; b = 0.97, SE = 0.54, p = .077, d = .47). In contrast, in the support first group, the vertical spread was significantly smaller in the first session (M = 10.7, SD = 4.23) compared to the second session (M = 11.7, SD = 4.03; b = -1.07, SE = 0.51, p = .042, d = -.51) (Figure 9A).

Horizontal Spread: For the horizontal spread of mechanical hypersensitivity, we again found a significant number x order interaction (F(1, 59.24) = 5.29, p = .025, ηp^2 = 0.082) such that in the alone first group, the horizontal spread was significantly larger in the first session (M = 4.10, SD = 1.72) than in the second session (M = 3.65, SD = 1.47; b = 0.49, SE = 0.22, p = .027, d = .59). In contrast, in the support first group, there was no significant difference between the first session (M = 3.75, SD = 1.43) and the second session (M = 3.94, SD = 1.39; b = -0.20, SE = 0.21, p = .344, d = -.23) (Figure 9B).

Exploratory analysis of the effect of Order and Condition on pinprick ratings at T0

These analyses were performed separately for the data collected for [1] and for the current study.

Jaltare et al., 2023 (N = 37 couples)

Pinprick intensity: There was a significant Condition by Order interaction (F(1, 373.71) = 48.40, p < .001, ηp^2 = 0.115). In the Alone condition, baseline ratings were similar between the Alone-First group and the Support-First group (b = −2.45, SE = 5.00, p = .627, d = -.27). In contrast, in the Support condition, baseline ratings were significantly higher (b = −14.81, SE = 5.01, p = .0054, d = -1.68) for

participants who received the Support condition first compared to those who received it after the Alone condition.

Pinprick Unpleasantness: As with the intensity ratings, there was a significant Condition by Order interaction (F(1, 373.67) = 38.15, $p < .001$, $\eta p^2 = 0.093$). For the Alone condition, baseline ratings were not significantly different (b = −3.31, SE = 5.21, p = .529, d = -.37) between the Alone-First group and the Support-First group. In contrast, in the Support condition, baseline ratings were significantly higher (b = −14.23, SE = 5.22, p = .010, d = -1.62) for participants who received the Support condition first compared to those who received it after the Alone condition.

Present Study (N = 28 couples)

Pinprick intensity: There was a significant Condition by Order interaction (F(1, 302) = 50.41, $p < .001$, $\eta p^2 = 0.143$). In the Alone condition, there was no significant difference in the ratings (b = −1.74, SE = 5.74, p = .764, d = -.16) between the Alone-First group and the Support-First group. In contrast, in the Support condition, baseline ratings were significantly higher (b = −17.96, SE = 5.74, p = .004, d = -1.71) for participants who received the Support condition first compared to those who received it after the Alone condition.

Pinprick Unpleasantness: The analysis for the baseline unpleasantness ratings revealed a significant Condition by Order interaction (F(1, 302) = 50.08, $p < .001$, $\eta p^2 = 0.142$). In the Alone condition, there was no significant difference (b = −4.49, SE = 5.35, p = .409, d = -.53) between the Alone-First group and the Support-First group. In contrast, in the Support condition, baseline ratings were significantly higher (b = −17.51, SE = 5.35, p = .0029, d = -2.07) for participants who received the Support condition first compared to those who received it after the Alone condition.

**Baseline Pinprick Intensity Ratings**		
**Jaltare et al., 2022 (N = 37 couples)**		
	**Support**	**Alone**
**Alone First**	14.39 (13.37)	22.85 (17.24)

**Support First**	**29.99 (17.56)**	23.73 (16.76)
**Present Study (N = 28 couples)**		
	**Support**	**Alone**
**Alone First**	14.82 (12.95)	21.60 (14.91)
**Support First**	**32.79 (22.71)**	23.33 (18.38)
**Baseline Pinprick Unpleasantness Ratings**		
**Jaltare et al., 2022 (N = 37 couples)**		
	**Support**	**Alone**
**Alone First**	11.81 (11.82)	18.46 (16.98)
**Support First**	**26.92 (19.62)**	21.08 (18.36)
**Present Study (N = 28 couples)**		
	**Support**	**Alone**
**Alone First**	6.46 (7.50)	10.32 (10.96)
**Support First**	**23.98 (23.47)**	14.81 (16.29)

Table S1: Mean and standard deviation of the Baseline (T0) Pinprick intensity and unpleasantness ratings, separately for the subsample from Jaltare et al., 2023 and the additional subsample collected as part of this study.

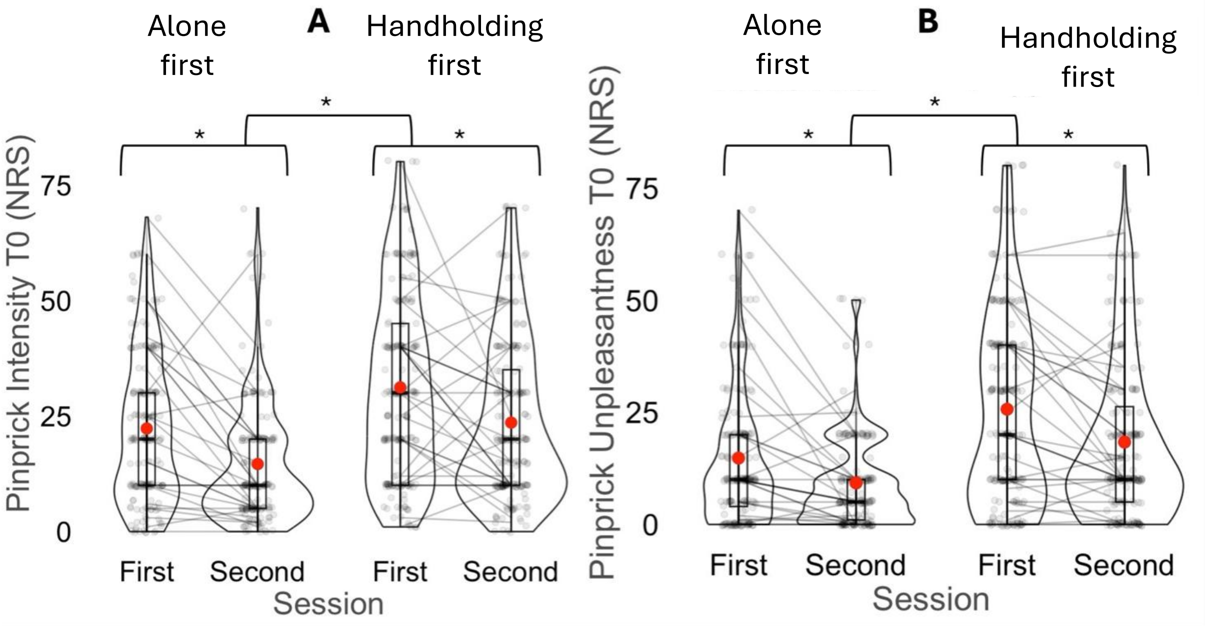

Figure S1: *Pinprick intensity and unpleasantness ratings at baseline (T0) by session and order group.* A) Raw pinprick intensity ratings (NRS) in response to pinprick stimulation, split by Session (first vs. second) and order group (alone first vs. support first). B) Raw pinprick unpleasantness ratings (NRS) for the same participants and sessions. Red dots represent group means; boxplots depict the interquartile range; violins illustrate the kernel density of the distribution. Grey lines connect individual participants across sessions to show within-subject change. Asterisks indicate statistical significance at *p* < .05.

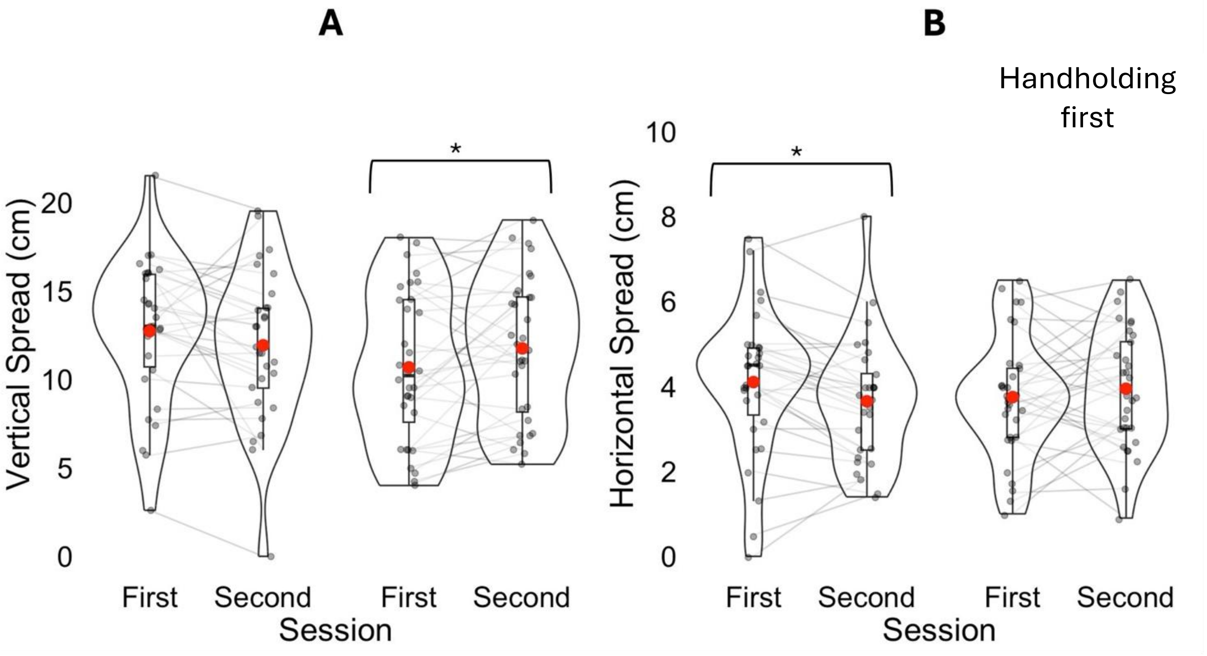

Figure S2: *Vertical and horizontal spread of mechanical hypersensitivity by session and order group.* A) vertical spread of hypersensitivity (cm) for each participant, split by session (first vs. second) and order group (alone first vs. handholding first). B) Raw horizontal spread of hypersensitivity (cm) for the same participants and sessions. Red dots indicate group means; boxplots show the interquartile range; violins illustrate the kernel density of the distribution. Grey lines connect individual participants across sessions to show within-subject change. Asterisks indicate statistical significance at *p* < .05.

Interaction between Order of support and Session number for MFS ratings

MFS Intensity Ratings

We found significant main effects of Number ($F(1, 1409.97) = 55.34$, $p < .001$, $\eta p^2 = 0.038$) and Order ($F(1, 61.22) = 5.27$, $p = .025$, $\eta p^2 = 0.079$), as well as a significant Number by Order interaction ($F(1, 1409.97) = 5.85$, $p = .016$, $\eta p^2 = 0.004$). Follow-up analyses revealed that for participants who

experienced the Alone condition first, MFS intensity ratings were significantly higher in the first session ($M = 73.4$, $SD = 16.1$) compared to the second measurement ($M = 66.6$, $SD = 16.6$) ($b = 5.50$, $SE = 0.81$, $p < .001$, $d = .51$). Similarly, when participants experienced the Support condition first, MFS intensity ratings were also higher for the first measurement ($M = 80.0$, $SD = 10.9$) compared to the second measurement ($M = 76.6$, $SD = 14.9$) ($b = 2.80$, $SE = 0.77$, $p = .0003$, $d = .26$), however the difference was smaller, as can be seen from the estimated coefficients.

MFS Unpleasantness Ratings

We found significant main effects of Number ($F(1, 1409.65) = 62.66$, $p < .001$, $\eta p^2 = 0.043$) and Order ($F(1, 61.23) = 5.50$, $p = .022$, $\eta p^2 = 0.082$), as well as a significant Number by Order interaction ($F(1, 1409.65) = 4.49$, $p = .034$, $\eta p^2 = 0.003$). Follow-up analyses revealed that when participants experienced the Alone condition first, MFS unpleasantness ratings were significantly higher for the first session ($M = 70.8$, $SD = 21.0$) compared to the second session ($M = 62.7$, $SD = 19.4$) ($b = 6.24$, $SE = 0.90$, $p < .001$, $d = .52$). Similarly, when participants experienced the Support condition first, unpleasantness ratings were also higher for the first session ($M = 79.1$, $SD = 12.4$) compared to the second session ($M = 74.9$, $SD = 18.1$) ($b = 3.60$, $SE = 0.86$, $p < .001$, $d = .30$), however, the difference was smaller when the support condition was experienced first.

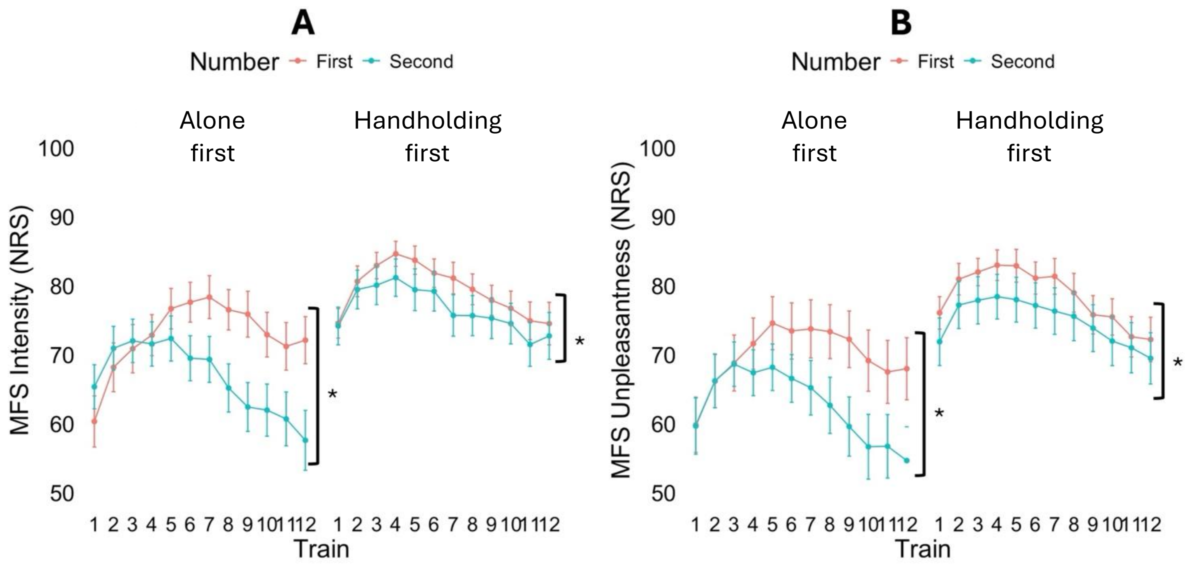

Figure S3: Mean MFS intensity and unpleasantness ratings across trains, split by session number (first vs second session) and order group. A) Mean MFS intensity ratings (NRS) across 12 trains of stimulation, shown separately for the First and Second sessions and for each Order group (Alone First vs. Handholding First). B) Mean MFS unpleasantness ratings (NRS) for the same participants and conditions. Error bars represent ±1 standard error of the mean. Brackets indicate significant main effects of session number ($p < .05$) within each order group.

Full Model Outputs for all analyses

Effect	Sum Sq	Mean Sq	NumDF	DenDF	F value	Pr(>F)	Signif
Condition	662.15	662.15	1.00	1409.97	5.85	0.02	*
Order	596.87	596.87	1.00	61.22	5.27	0.03	*
Condition :Order	6267.80	6267.80	1.00	1409.97	55.34	0.00	***

Table S2: MFS Intensity Ratings: Full outputs of the following model: *MFS Intensity ~ Condition * Order + (1|Participant)*

Effect	Sum Sq	Mean Sq	NumDF	DenDF	F value	Pr(>F)	Signif

Condition	631.46	631.46	1.00	1409.65	4.49	0.03	*
Order	774.08	774.08	1.00	61.23	5.50	0.02	*
Condition :Order	8812.70	8812.70	1.00	1409.65	62.66	0.00	***

Table S3: MFS Unpleasantness Ratings: Full outputs of the model: *MFS Unpleasantness ~ Condition * Order + (1|Participant)*

Effect	Sum Sq	Mean Sq	NumDF	DenDF	F value	Pr(>F)	Signif
T0	8469.51	8469.51	1.00	597.62	49.64	0.00	***
Condition	10.46	10.46	1.00	655.02	0.06	0.80	
Arm	17123.70	17123.70	1.00	652.66	100.37	0.00	***
Order	1441.09	1441.09	1.00	48.63	8.45	0.01	**
Condition :Arm	1264.53	1264.53	1.00	654.23	7.41	0.01	**
Condition :Order	1121.36	1121.36	1.00	684.84	6.57	0.01	*
Arm:Orde r	4240.37	4240.37	1.00	653.36	24.85	0.00	***
Condition :Arm:Ord er	177.23	177.23	1.00	652.53	1.04	0.31	

Table S4: Pinprick Intensity Ratings: Full outputs of the model: *T1 Pinprick Intensity ~ T0 + Condition*Arm*Order + (1|Participant)*

Effect	Sum Sq	Mean Sq	NumDF	DenDF	F value	Pr(>F)	Signif
Condition	23.65	23.65	1.00	1418.75	0.15	0.70	
Arm	9288.68	9288.68	1.00	1415.48	60.10	0.00	***

New_Time	11064.44	11064.44	1.00	1417.53	71.59	0.00	***
Condition:Arm	65.58	65.58	1.00	1415.48	0.42	0.51	
Condition:New_Time	21.05	21.05	1.00	1417.58	0.14	0.71	
Arm:New_Time	7851.40	7851.40	1.00	1415.48	50.80	0.00	***
Condition:Arm:New_Time	1199.70	1199.70	1.00	1415.48	7.76	0.01	**

Table S5: Pinprick Intensity: Full outputs of the model: *Pinprick Intensity ~ Condition*Arm*Time + (1|Participant)*

Effect	Sum Sq	Mean Sq	NumDF	DenDF	F value	Pr(>F)	Signif
T0	2063.74	2063.74	1.00	609.85	12.16	0.00	***
Condition	242.78	242.78	1.00	652.70	1.43	0.23	
Arm	17574.96	17574.96	1.00	650.55	103.53	0.00	***
Order	522.23	522.23	1.00	46.65	3.08	0.09	
Condition:Arm	262.30	262.30	1.00	649.89	1.55	0.21	
Condition:Order	630.05	630.05	1.00	679.59	3.71	0.05	
Arm:Order	7088.14	7088.14	1.00	651.07	41.75	0.00	***
Condition:Arm:Order	658.20	658.20	1.00	649.86	3.88	0.05	*

Table S6: Pinprick Unpleasantness Ratings: Full outputs of the model: *T1 Pinprick Unpleasantness ~ T0 + Condition*Arm*Order + (1|Participant)*

Effect	Sum Sq	Mean Sq	NumDF	DenDF	F value	Pr(>F)	Signif
Condition	31.72	31.72	1.00	58.87	7.49	0.01	**

Order	4.50	4.50	1.00	59.64	1.06	0.31	
Condition:Order	0.07	0.07	1.00	58.87	0.02	0.90	

Table S7: Vertical Spread: Full outputs of the model: *Vertical Spread ~ Condition*Order + (1|Participant)*

Effect	Sum Sq	Mean Sq	NumDF	DenDF	F value	Pr(>F)	Signif
Condition	3.60	3.60	1.00	59.24	5.29	0.02	*
Order	0.00	0.00	1.00	59.95	0.00	0.98	
Condition:Order	0.66	0.66	1.00	59.24	0.96	0.33	

Table S8: Horizontal Spread: Full outputs of the model: *Horizontal Spread ~ Condition*Order + (1|Participant)*

Exploratory Analyses

Effect of Order and condition on baseline pinprick ratings at T0

Effect	Sum Sq	Mean Sq	NumDF	DenDF	F value	Pr(>F)	Signif
Order	585.37	585.37	1.00	61.86	6.30	0.01	*
Arm	24.87	24.87	1.00	679.86	0.27	0.61	
Condition	9.83	9.83	1.00	682.10	0.11	0.75	
Order:Arm	168.01	168.01	1.00	679.86	1.81	0.18	
Order:Condition	8997.58	8997.58	1.00	682.10	96.81	0.00	***
Arm:Condition	342.48	342.48	1.00	679.86	3.68	0.06	
Order:Arm:Condition	0.02	0.02	1.00	679.86	0.00	0.99	

Table S9: **P**inprick Intensity: Full outputs of the model: T0 Ratings ~ Order*Arm*Condition + (1|Participant)

Effect	Sum Sq	Mean Sq	NumDF	DenDF	F value	Pr(>F)	Signif
Condition	191.67	191.67	1.00	681.69	2.57	0.11	
Arm	103.26	103.26	1.00	679.94	1.38	0.24	
Order	524.36	524.36	1.00	61.95	7.03	0.01	*
Condition:Arm	4.36	4.36	1.00	679.94	0.06	0.81	
Condition:Order	6332.96	6332.96	1.00	681.69	84.93	0.00	***
Arm:Order	222.87	222.87	1.00	679.94	2.99	0.08	
Condition:Arm:Order	0.63	0.63	1.00	679.94	0.01	0.93	

Table S10: Pinprick Unpleasantness: Full outputs of the model: T0 Ratings ~ Order*Arm*Condition + (1|Participant)

Exploratory analysis of the combined effect of Order and Session Number

Effect	Sum Sq	Mean Sq	NumDF	DenDF	F value	Pr(>F)	Signif
Number	0.07	0.07	1.00	58.87	0.02	0.90	
Order	4.50	4.50	1.00	59.64	1.06	0.31	
Number:Order	31.72	31.72	1.00	58.87	7.49	0.01	**

Table S11: Vertical Spread: Full outputs of the model: Vertical Spread ~ Number*Order + (1|Participant)

Effect	Sum Sq	Mean Sq	NumDF	DenDF	F value	Pr(>F)	Signif
Number	0.66	0.66	1.00	59.24	0.96	0.33	
Order	0.00	0.00	1.00	59.95	0.00	0.98	
Number:Order	3.60	3.60	1.00	59.24	5.29	0.02	*

Table S12: Horizontal Spread: Full outputs of the model: Horizontal Spread ~ Number*Order + (1|Participant)

Exploratory analysis of the effect of Order and Condition on pinprick ratings at T0

Jaltare et al., 2023 (N = 37 couples)

Effect	Sum Sq	Mean Sq	NumDF	DenDF	F value	Pr(>F)	Signif
Order	238.71	238.71	1.00	33.88	3.07	0.09	
Arm	70.95	70.95	1.00	371.87	0.91	0.34	
Condition	218.79	218.79	1.00	373.71	2.82	0.09	
Order:Arm	32.12	32.12	1.00	371.87	0.41	0.52	
Order:Condition	3759.58	3759.58	1.00	373.71	48.40	0.00	***
Arm:Condition	0.22	0.22	1.00	371.87	0.00	0.96	
Order:Arm:Condition	37.39	37.39	1.00	371.87	0.48	0.49	

Table S13: Pinprick intensity: Full outputs of the model: T0 Intensity ~ Order*Arm*Condition + (1|Participant)

Effect	Sum Sq	Mean Sq	NumDF	DenDF	F value	Pr(>F)	Signif
Condition	9.51	9.51	1.00	373.67	0.12	0.73	
Arm	123.61	123.61	1.00	371.99	1.61	0.21	
Order	223.68	223.68	1.00	34.00	2.91	0.10	
Condition:Arm	30.64	30.64	1.00	371.99	0.40	0.53	
Condition:Order	2934.06	2934.06	1.00	373.67	38.15	0.00	***
Arm:Order	136.29	136.29	1.00	371.99	1.77	0.18	
Condition:Arm:Order	0.00	0.00	1.00	371.99	0.00	1.00	

Table S14: Pinprick Unpleasantness: Full outputs of the model: T0 Unpleasantness ~ Order*Arm*Condition + (1|Participant)

Present Study (N = 28 couples)

Effect	Sum Sq	Mean Sq	NumDF	DenDF	F value	Pr(>F)	Signif
Order	336.38	336.38	1.00	26.00	3.07	0.09	
Arm	4.53	4.53	1.00	302.00	0.04	0.84	
Condition	150.67	150.67	1.00	302.00	1.37	0.24	
Order:Arm	181.57	181.57	1.00	302.00	1.66	0.20	
Order:Condition	5529.07	5529.07	1.00	302.00	50.41	0.00	***
Arm:Condition	783.24	783.24	1.00	302.00	7.14	0.01	**
Order:Arm:Condition	32.81	32.81	1.00	302.00	0.30	0.58	

Table S15: Pinprick Intensity: Full output of the model: T0 Intensity ~ Order*Arm*Condition + (1|Participant)

Effect	Sum Sq	Mean Sq	NumDF	DenDF	F value	Pr(>F)	Signif
Condition	592.01	592.01	1.00	302.00	8.32	0.00	**
Arm	6.86	6.86	1.00	302.00	0.10	0.76	
Order	309.55	309.55	1.00	26.00	4.35	0.05	*
Condition:Arm	86.01	86.01	1.00	302.00	1.21	0.27	
Condition:Order	3562.01	3562.01	1.00	302.00	50.08	0.00	***
Arm:Order	88.05	88.05	1.00	302.00	1.24	0.27	
Condition:Arm:Order	0.58	0.58	1.00	302.00	0.01	0.93	

Table S16: Pinprick Unpleasantness: Full output of the model: T0 Unpleasantness ~ Order*Arm*Condition + (1|Participant)

Supplementary references

[1] Jaltare KP, Vanderijst L, Karos K, Torta DM. The impact of the social context on the development of secondary hyperalgesia: an experimental study. Pain 2023;164(12):2711-2724.
